# Investigating the Effectiveness of Using a Situated Simulation-Based Program to Improve Occupational Therapy Students' Interactions and Observation Skills with Children

**DOI:** 10.1155/2021/1698683

**Published:** 2021-11-03

**Authors:** Chia-Hui Hung, Tzu-Hua Ho, Chen-Yung Lin

**Affiliations:** ^1^Department of Occupational Therapy, Chung Shan Medical University and Chung Shan Medical University Hospital, Taichung City 40201, Taiwan; ^2^Department of Early Childhood Education, Asia University, Taichung 41354, Taiwan; ^3^Graduate Institute of Science Education, National Taiwan Normal University, Taipei 106, Taiwan

## Abstract

**Purpose:**

Interaction and observation are critical skills for occupational therapists who work with pediatric clients. The objective of this study was to investigate whether using standardized child patients within a situated simulation-based (SSB) program increases students' knowledge and clinical skills when working with children in occupational therapy.

**Materials and Methods:**

This controlled trial with multiple measures recruited students from the pediatric occupational therapy curriculum enrolled in an SSB program in consecutive academic years (*n* = 62). Experimental group students participated in a simulation experience with video training sessions, followed by an SSB exam with standardized child patients; the control group performed the video training simultaneously. Quantitative outcomes included quizzes to measure clinical knowledge, video training scores, and a situated simulation exam to assess clinical skills.

**Results:**

The experimental group had a significantly higher postwritten quiz scores than the control group; the video training scores were not significantly different between groups. Linear regression analysis showed a significant association between the SSB exam and postwritten quiz scores (*β* = 0.487, *p* = 0.017). The experimental group had a total pass rate of 65.6% for the SSB exam. The communication and interaction pass rate was 53.1%; the basic evaluation rate was 68.8%, implying that communication/interaction skills are hard to simulate from video training alone; therefore, the authentic fidelity of the SSB program needs to improve further to enhance learning.

**Conclusions:**

The SSB program with standardized child patients improved students' clinical knowledge and skills more than lectures and practice alone. Using standardized child patients in programs or exams appears to positively influence students' performance. Situated simulation-based learning that allows the realistic practice of observation and communication skills may enhance students' clinical competency. Future research should develop standard training methods and evaluation processes in high-fidelity simulations for generalized use in other occupational therapy programs.

## 1. Introduction

Simulation-based education has been well established for many decades in curricula for health professionals. Much evidence suggests that in order to enhance their competency and clinical reasoning, students must be provided with an effective learning environment that provides authentic and context-specific clinical situations to develop professional knowledge, skills, attitudes, and experience [1–4]. Three elements of critical simulations can support learning and facilitate situated cognition: (1) task trainers, mannequins, or standardized patients; (2) situated simulation of authentic environments such as a ward or clinical environment; and (3) the use of equipment, videos, or interactive virtual reality programs to recreate the clinical reasoning and actions of specific practice situations [5–8]. These simulation elements can be used and arranged in a task-based learning program that incorporates multiple, changing scenarios in a learning environment based on the content and fidelity of the teaching and learning activities [1].

Situated simulation-based (SSB) programs include an authentic environment that imitates some real thing, patient, or clinical process [9], while the materials (objects, texts, technologies, and settings) are tailored to specific practice and learning objectives to create high-fidelity experiences [10]. Accumulating evidence has shown that practices related to standardized patient simulations enhance students' learning outcomes and clinical reasoning skills by enabling the students to experience decision-making in real time [2]. However, the literature on simulation in occupational therapy and the evidence supporting its use and effectiveness in the classroom are limited to a few published articles [11–16]. These articles examined simulated occupational therapy experiences related to physical disability and psychiatric disorders. However, less attention has been paid to simulation in pediatric occupational therapy education; this may be due to issues of ethics, reliability, and consistency when using children as standardized patients. Moreover, utilizing children as standardized patients is more expensive and time-consuming than using adults [17–19]. However, evidence indicates that using children as standardized patients were perceived as fair, acceptable, realistic, and feasible by all stakeholders and were concluded to confer a positive educational impact [20]. Occupation therapy students are relatively young themselves; thus, they may find it difficult to identify as therapists who need to interact objectively with children; therefore, students can often feel unprepared and challenged when treating children in their fieldwork practice. Based on the professional requirements of occupational therapy, students at this stage of education require more opportunities to acquire the skills of observation, communication, and interaction and even to play with children during evaluation or therapeutic activities. Therefore, a repeatable and harmless form of practice needs to be established for the use of children as standardized patients [21]. However, no such methods of practicing interaction or communication skills have yet been provided by simulators.

According to the normalization process theory proposed by Springfield et al. [4], effective simulation-based education requires the development of a set of programs that are standardized and routinely and sustainably embedded in learning. Other methods for simulation-based education may also provide alternative strategies. For example, including videos in simulation-based training may be an attractive method for reducing the costs of simulation-based medical education, without compromising learning [8, 22]. Both video-based and standardized patients represent suitable simulation methods for technical training in the classroom [23]; however, video-based training has lower simulation fidelity than standardized child patient training methods. Therefore, any combination of these approaches to create the best learning outcomes must be supported by evidence.

The purpose of the present study was to investigate the effectiveness of using standardized child patients to develop a situated simulation-based program to increase students' knowledge and clinical skills when working with children in occupational therapy. This work was designed to address the following research questions: first, do the students who participate in the SSB program perform better than their counterparts who only receive video training? Second, does the SSB exam reflect what students learn in the SSB program?

## 2. Materials and Methods

### 2.1. Research Design

This cohort study employed quantitative methods to obtain a holistic understanding of the efficacy of the simulation-based program. Guided by previous research results [24], the SSB program was designed to include lectures, practice, and exams to fully encompass the essence of simulation-based education. In the first step, the reliability and validity of the SSB program were validated. Then, the performance of the control and experimental groups of students was compared. A linear regression model was established based on the video-based training scores, written quizzes, and SSB exam scores to interpret the performance of the students after the SSB program. Finally, the essential characteristics of the SSB exam are presented to illustrate the participants' performance. Ethical approval for the protocol of this study was granted by the internal review committee of Fu Jen Catholic University (project no. C103107). At the initial introduction to the program, the students were informed that their participation was voluntary, and oral consent was obtained from all of the participating students and educators.

### 2.2. Participants

This nonrandomized controlled trial employed multiple outcome measures. Sixty-two fourth-year occupational therapy students enrolled in the pediatric occupational therapy program with no prior clinical experience were included. The participants (26 males, 36 females) ranged in age from 19 to 20 years (mean = 19.6 years). Participants who volunteered to participate in the SSB exam were assigned to the experimental group (*n* = 30). Therefore, the other participants who did not take the SSB exam were assigned to the control group (*n* = 32). Quantitative data were collected, including the students' scores on written quizzes administered before and after the SSB program and the students' scores for observations of video-based training evaluated by the class teachers. A SSB exam was administered and collected after the students completed the SSB program. Two occupational therapy teachers served as the primary evaluators and provided feedback communication regarding the performance of all participants.

### 2.3. Procedures

The SSB program was a 17-week program with three parts. The first part was lectures, including ten weeks of lectures introducing theoretical knowledge on human development and clinical evaluation skills. In this learning component, the participants were also guided to practice their interaction and observation skills using role play techniques.

The second part was a four-week simulation training program intended to give students practice with observations. The simulation sessions consisted of a four-hour course that included two video-based simulations. Students were guided to observe realistic detail about children's play in the child play video by using a clinical observation form with seven developmental domains (gross motor, fine motor, visual perception, cognition/communication, social interaction, and other behaviors). After the video simulations, the clinical observation forms were sent back to the teacher, who evaluated the students' observation findings and provided the scores. The final debriefing was provided after the video simulations via real-time instructor feedback to discuss the scenarios and evaluate the students' observation capabilities.

**Figure 1:**
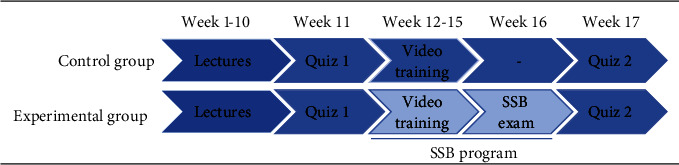
Timeline of the study for the students in the experimental and control groups.

The third part included three evaluations of the program. The pre- and post-SSB programs' written quizzes were administered in the 11th and 17th weeks of the academic year to examine the knowledge and clinical skills acquired by the students during the semester's learning. In the 16th week, a two-station SSB exam for clinical skills was administered. The study timeline for the students in the experimental and control groups is shown in Figure 1. The various skills assessed are listed in Table 1.

### 2.4. Simulations

For this study, the SSB program was defined as exposure to the full video training and participation in the SSB exam. To participate in the entire SSB program, students must take the clinical skill training program and then complete a clinical skill exam.

Two occupational therapy teachers developed the scenarios in the high-fidelity videos in the SSB program to meet the training goals and skill requirements. Two training videos were created, including child play and social interaction. Each video was approximately 10 minutes long. A clinical observation form was designed to allow students to practice making observations.

The SSB exam was developed to assess clinical skills during the final exam. A child was employed as the standardized patient and was trained to portray the adopted patient's medical history and specific case scenario. The simulation scenarios were constructed to mimic the clinical activities and environment in clinical settings, such as clinical treatment rooms. The case was reflective of a representative noncritical patient requiring occupational assessment and intervention. Students were encouraged to interact with the standardized patient in a similar manner as they would with actual pediatric patients. The raters at the stations acted as passive evaluators and did not guide or prompt the students.

To ensure the high fidelity of the SSB exam, a situated simulation therapeutic room was prepared (about 7∗10 square meters). Similar to an actual pediatric occupational therapy room, the room was covered with a thick mat to prevent injury due to falls, and therapeutic equipment, including a ball pool, suspension system, wedges, rollers, and balls, was placed on the mat. A small desk was prepared for the standardized child patient in the middle of the room, who were five-year-old kindergarten-attending children with typical development. Before the exam, the standardized child patients and their parents provided informed consent to participate. Then, an examiner introduced the exam and the clinical task(s) to be carried out to the child and their parents.

The exam included two connected stations and was staffed with a standardized patient to evaluate the students' communication, observation, and interaction skills (station 1) and basic evaluation skills (station 2). A checklist with twelve items was developed. Each item was scored on a three-point rating scale (0—“not completed”; 1—“partially completed”; and 2—“completed”) with descriptive anchors to provide a scoring rubric for the scale. A five-point overall score (1—“unqualified”; 2—“borderline”; 3—“pass”; 4—“good”; and 5—“excellent”) was also applied to evaluate students' overall performance at each station. The students were asked to imagine that they were newly qualified occupational therapists who had been tasked with evaluating a child. Each student had 10 minutes to read the exam scenario outside the station. Then, each student was given 10 minutes at each station to interact with the standardized patient at the station. Overall, each student spent 30 minutes completing the exam.

An expert group of four occupational therapy teachers was asked to review the content for the stations and the checklists with a pro forma using a Likert-type scale to establish the content validity. The factors reviewed were intended to clarify whether the rubric included the essential steps linked to the exam content and covered the essential skills for an occupational therapy student, whether the exam content was in line with the simulation-based curriculum, whether the settings and contexts of the stations were authentic, and whether the instructions to the examinees were unambiguous. Based on the expert feedback, revisions were made to remove ambiguity and develop a consistent scoring system to distinguish students' clinical skill levels. Finally, the scoring rubric for the simulation exam was considered a valid measurement of student performance, as previously described [16].

The item-total correlation between the students' scores at the two stations and their total scores in the examination was calculated to assess reliability and validity. A station with a correlation coefficient of >0.7 was considered to have good reliability. The reliability (internal consistency) of the examination was assessed using Cronbach's *α* to measure the stability of the different stations. Cronbach's *α* was calculated using the total scores in the examination. A value > 0.8 was considered to indicate good reliability and appropriate for educational research [25]. Moreover, two raters were employed to evaluate the interrater reliability; the rater at each station graded the students' clinical skills and performance according to a given set of checklists and a scoring rubric. After the exam, another rater watched a recording of the exam and completed the second rating to establish interrater reliability.

### 2.5. Data Collection and Statistical Analysis

Data collection included the students' scores on the written quizzes, observation forms, and checklists. The marks were tabulated for data analysis in a deidentified format and analyzed using SPSS software (IBM, Armonk, NY, USA) to complete the three parts of the statistical analysis. First, to assess the differences between the experimental and control groups, two-sample (independent) *t*-tests were conducted to determine if the students' mean quiz scores differed between groups (*n* = 62); then, the paired *t*-tests were used to determine if the mean quiz and video training scores differed between the pre- and post-SSB programs (*n* = 30, *n* = 32). Second, to perform an in-depth assessment of the effect of the SSB on learning, a linear regression model was used to determine whether the quizzes and video training were predictors of the experimental group's results in the SSB exam (*n* = 30). Finally, descriptive statistics were used to present the experimental group's pass rates and performance scores in the SSB exam (*n* = 30).

## 3. Results

### 3.1. Properties of the SSB Exam

The assessment of content validity showed that the I-CVIs were 0.98 and 0.85 at the item level; the S-CVI of the overall checklist was 0.93 at the scale level, which was acceptable (Table 2). The evaluation of reliability revealed that the internal consistency, calculated using Cronbach's *α*, was 0.88. The interrater reliabilities for the stations, calculated using Pearson's correlation coefficient *r*, were 0.95 and 0.83. The validity and reliability tests indicated that the situated simulation exam checklist was suitable as a formal scoring rubric for evaluating clinical skills in the situated simulation course. The difficulty indexes (*P*) of the two stations were 1 and 0.2, respectively, and the discriminatory indexes of the stations were 0.53 and 0.91, respectively. These results imply that the first station was more complex than the second station. The overall mean percentage score for this examination was 42.31% (Table 3). The average score for all stations in this examination was moderately acceptable (0.5).

### 3.2. Intervention Effect of the SSB Program

**Figure 2:**
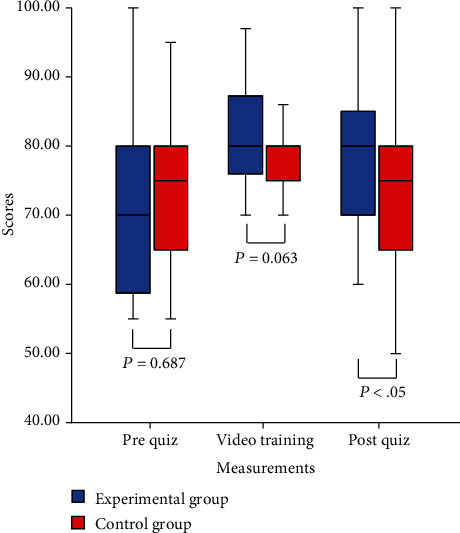
Boxplots of the prequiz scores, video training scores, and postquiz scores for the experimental and control groups.

An independent sample *t*-test was conducted to evaluate the research question: did the students who participated in the SSB program perform better than their counterparts who did not complete the SSB program? The mean scores on the pre-SSB program written quizzes were 72.33 ± 13.57 for the experimental group and 73.59 ± 10.87 for the control group. The differences were not significant, showing that the two groups had an equal baseline (*t* (60) = −0.405, *p* = 0.687). The mean scores on the post-SSB program written quizzes were 79.33 ± 10.56 for the experimental group and 72.66 ± 13.97 for the control group. The tests revealed a moderate effect size for the SSB program (*t* (60) = 2.112, *p* = 0.039, Cohen′s *d* = 0.62). The mean score on the post-SSB program written test for the experimental group increased from 72.33 ± 13.57 before the SSB intervention to 77.93 ± 10.56 after the SSB intervention (*t* (29) = −3.068, *p* = 0.005). The SSB program significantly improved the quiz scores and exam results of the students in the experimental group. These results are summarized and presented in Figure 2.

### 3.3. Effect of the SSB Program on Development of Communication Skills and Basic Evaluation Skills

To identify what students learned in the SSB program, we used descriptive statistics to assess the scores of the experimental group (*n* = 30) in the SSB exam. The characteristics of each station in the situated simulation exam are shown in Table 4. The average scores at the stations were 12.5 ± 1.3 for interaction skills and 8.2 ± 1.2 for basic evaluation skills. When the borderline group method was applied as the standard-setting method [26], 17 participants passed and 13 failed the exam. The pass rates for the two stations were 53.1% and 68.8%, respectively, and the overall pass rate was 65.6%.

### 3.4. Contribution of the SSB Exam to Students' Performance

Multiple linear regression analysis showed that the correlations between the variables (video training score, post-SSB program written quizzes, and SSB exam) were statistically significant (*R*^2^ = .211, *F* (2, 27) = 3.60, *p* = 0.041 < 0.05). The SSB exam accounted for 48.7% (*β* = .487, *t* = 2.541, *p* < 0.05) of the variance in the students' performance in their final written quizzes; however, the video training score did not significantly determine the students' performance in the final written quizzes (*p* = 0.705).

## 4. Discussion

SSB programs are aimed at creating authentic clinical situations to enable students to develop clinical skills and improve the safety of clinical practice. The main aims of this study were to investigate the effectiveness of the SSB program with standardized child patients and evaluate the students' performance in the SSB program. Students who participated in the SSB program had significantly higher accuracy in the post-SSB program written quizzes than the control group who did not participate in the SSB exam; however, the video training scores were not significantly different between the two groups. Students who participated in the SSB program performed better in the post-SSB program quiz. However, the students' communication and interaction pass rates in the SSB exam indicated that the application of the video training alone did not help the students to develop communication/interaction skills. Overall, the SSB program with standardized child patients helps students to better integrate and apply their learning, experience, and clinical skills to knowledge more than lectures and practice alone.

### 4.1. The SSB Program Integrates Declarative Knowledge and Procedural Skills

The students in both groups had significantly better scores on the post-SSB program written quiz than on the pre-SSB program written quiz, indicating the importance of lectures to transmit clinical knowledge. However, the students memorized and used declarative knowledge to achieve high scores, which is rote learning. Declarative knowledge refers to facts or information stored in the memory that is considered static and more straightforward to verbalize than procedural knowledge. In contrast, the performance outcomes of medical professionals rely on procedural knowledge, which is emphasized by the medical profession, and cannot be evaluated by a written test. Ideally, the outcome measures of simulation-based education should be assessed on real patients or standardized patients to confirm the transfer of learning [21]. The SSB program in the present study provided students with a chance to practice procedural skills in a simulated clinical situation and integrate the knowledge they had learned to develop competency, representing mastery learning, with competence arising from experience.

### 4.2. A Real Social Environment Is Essential for Developing Nontechnical Skills

Station 1 (communication and interaction) in this study was more challenging than station 2 (basic assessment skills). It appears that evaluation competencies are categorized as technical competencies, for which standard procedures can be followed, and repetition could refine students' knowledge of procedures. However, communication and interaction are nontechnical skills, and the literature suggests that practice in situated simulations enhances students' clinical decision-making skills, satisfaction, and self-efficacy. For this reason, the performance of nontechnical competencies could be improved [27, 28].

The SSB program employed in this study improved the students' skills related to evaluating children. However, the results of the experimental group in the SSB exam indicate that the current video training is not sufficiently effective for developing students' communication and interaction skills. Provision of high-fidelity simulations with physical and social environments similar to actual clinical situations, such as using role play and standardized patients, has been suggested to improve the level of simulation and provide an authentic interaction experience. It is noteworthy that a postsimulation debriefing [22, 29], which is a high-quality debriefing and reflection process following the training, is the most critical element of improving communication and interaction skills. A learning environment with high physical fidelity that provides opportunities to practice, debrief, and apply knowledge can help students to develop clinical skills. Also, cognitive fidelity, such as wearing uniforms and carrying identification, can raise students' awareness of professional behavior and ultimately enhance the authenticity of the experience [5].

### 4.3. Standardized Child Patients Benefit to the Professional Learning in Occupational Therapy

Children's voices are gaining more attention and respect in clinical practice. However, issues related to involving standardized child patients in medical education training programs have seldom been addressed. Some healthcare professions, such as dentistry and nursing, have recently used standardized child patients in their professional clinical skill simulation training, as these professionals provide many services for children [30, 31]. Occupational therapy also provides services for children. Therefore, content that focuses on interacting with children is a valuable component of the learning process for a pediatric occupational therapy curriculum. We suggest that simulators or videos should be incorporated into training programs to train students in skills such as observation and evaluation. Including standardized child patients in the objective structured clinical examination would help to enhance students' communication skills and relationship-building learning [15, 32].

In terms of the training of standardized child patients, first, the children should preferably be over five years old and have already attended kindergarten, which has been shown to help to make it easier for children to follow instructions. Second, preparing specific concrete objects for operating in the exam situation that trigger the standardized child patient to connect the test scenario and process, such as paper and pencils, will help to ensure that the standardized child patient connection addresses what needs to be answered; for example, “prepare the ball” is connected to the action that needs to be performed. Third, several rehearsal sessions are needed, and after each rehearsal, the child must be gently reminded, assisted, and encouraged to improve their next performance. These training methods can be used to complete the training of standardized child patients and provide suggestions for future occupational therapy curriculum teaching and learning.

The present study has several limitations, including the quasiexperimental study design and the fact that certain confounding factors could not be excluded, including the growth factor of the students. Also, the SSB exam was embedded in a specific program curriculum; therefore, it was not possible to compare the performance of the students with students who had participated in different, but complementary, educational activities. Generalization of the results to other occupational therapy programs may not be possible without further study.

## 5. Conclusions

The SSB program with standardized child patients improved students' clinical knowledge and skills more than lectures and practice alone. Using simulation-based learning to allow realistic practice of evaluation and communication skills may ultimately enhance students' clinical competency when interacting with children. Therefore, the learning objectives for simulation-based learning should include knowledge acquisition and clinical practice, and situated simulated learning experiences should be provided to strengthen these essential skills. The use of standardized child patients in simulation-based programs or exams appears to positively influence students' performance.

SSB programs may enhance clinical skills and improve patient care and safety in the rehabilitation domain. The key elements of successfully implementing a SSB program that facilitates learning are fidelity, course planning, scenarios with clear training goals, evaluation, and quality debriefing. To increase the effectiveness and efficiency of training, medical educators should focus on these factors when establishing situated simulation programs in their professional areas. The results of the present study may provide helpful information for future studies on simulation-based education in occupational therapy.

## Figures and Tables

**Table 1:** Content of the situated simulation-based exam.

Problem	Description	Simulation method
Communication skills	To test the relationship-building skills and interaction skills	Standardized child patients
Developmental examination skills	To test the skills needed to perform the evaluation, including guiding the child in the exam and executing the procedures of the evaluation	Standardized child patients
Professional knowledge	To test the knowledge of various human development sequences	Standardized child patients

**Table 2:** Reliability and validity of the situated simulation-based exam.

		Reliability		Validity	
Station	Cronbach's *α*	Item total correlation	Interrater reliability	S-CVI	I-CVI
1	0.88	0.89	0.95	0.93	0.98
2		0.87	0.83		0.85

Station 1: communication and interaction; station 2: basic evaluation skills.

**Table 3:** Overall analysis of the situated simulation-based exam.

	Overall score		Item analysis	
Station	Mean % score	Average score (SD)	Difficulty index (*P*)	Discrimination index
1	42.97	3.28 (0.73)	1	0.53
2		3.44 (0.56)	0.2	0.91

Station 1: communication and interaction; station 2: basic evaluation skills.

**Table 4:** Summary of students' scores at each station in the situated simulation-based exam.

	All participants (*n* = 32)							
Station	Scores		Overall rating (%)					Pass (%)	Overall pass rate (%)
	*M* ± SD	Range	1	2	3	4	5		
1	12.5 ± 1.3	10–14	0	6.3	40.6	21.9	21.3	53.1	65.6
2	8.2 ± 1.2	5–10	3.1	6.3	21.9	15.6	53.1	68.8	

Station 1: communication and interaction; station 2: basic evaluation skills. Scores: 0—not completed; 1—partially completed; and 2—completed. Overall ratings: 1—unqualified; 2—borderline; 3—pass; 4—good; and 5—excellent.

## Data Availability

The data used to support the findings of this study are available from the corresponding author upon request.
